# Chromosomal polymorphism and comparative chromosome painting in the rufous-collared sparrow (*Zonotrichia capensis*)

**DOI:** 10.1590/1678-4685-GMB-2017-0367

**Published:** 2018-11-14

**Authors:** Sandra Eloisa Bülau, Rafael Kretschmer, Ricardo José Gunski, Analía del Valle Garnero, Patricia C. M. O’Brien, Malcolm A. Ferguson-Smith, Edivaldo Herculano Correa de Oliveira, Thales Renato Ochotorena de Freitas

**Affiliations:** ^1^ Programa de Pós-graduação em Genética e Biologia Molecular (PPGBM), Universidade Federal do Rio Grande do Sul, Porto Alegre, RS, Brazil; ^2^ Programa de Pós-graduação em Ciências Biológicas (PPGCB), Universidade Federal do Pampa, São Gabriel, RS, Brazil; ^3^ Cambridge Resource Centre for Comparative Genomics, University of Cambridge, Cambridge, United Kingdom; ^4^ Instituto de Ciências Exatas e Naturais, Universidade Federal do Pará, Belém, PA, Brazil; ^5^ Laboratório de Cultura de Tecidos e Citogenética (SAMAM), Instituto Evandro Chagas, Ananindeua, PA, Brazil

**Keywords:** Birds, chromosomal rearrangements, molecular cytogenetics, FISH

## Abstract

*Zonotrichia capensis* is widely distributed in the Neotropics. Previous cytogenetic studies demonstrated the presence of polymorphisms in two chromosome pairs (ZCA2 and ZCA4). Here, we report results based on comparative chromosome painting, using probes derived from *Gallus gallus* and *Leucopternis albicollis*, focused on characterizing the chromosome organization of *Z. capensis*. Our results demonstrate the conservation of ancestral syntenies as observed previously in other species of passerine. Syntenies were rearranged by a series of inversions in the second chromosome as described in other Passeriformes, but in this species, by using probes derived from *L*. *albicollis* we observed an extra inversion in the second chromosome that had not previously been reported. We also report a paracentric inversion in pair 3; this chromosome corresponds to the second chromosome in *Zonotrichia albicollis* and may indicate the presence of ancestral inversions in the genus. The chromosomal inversions we found might be important for understanding the phenotypic variation that exists throughout the distribution of *Z*. *capensis*.

## Introduction

The rufous-collared sparrow (*Zonotrichia capensis*) is a common small Neotropical passerine. It occurs in open areas from Tierra del Fuego in Argentina to the province of Chiapas in Mexico and from sea level to 5,000 meters above sea level in the Andes Mountains (Sick, 1997; Rising and Jamarillo, 2011; Bird Life International, 2015). It is one of the most polytypic bird species, with more than 20 subspecies described, presenting variations in morphology, migratory behavior, and vocal dialects, which may be due to local adaptation or phenotypic plasticity (Chapman, 1940; Handford, 1983, 1985; Kopuchian *et al.*, 2004; Moore *et al.*, 2005; Cheviron *et al.*, 2008). However, even with this marked phenotypic variation, *Z. capensis* does not exhibit genetic structuring among its populations, although they present a pattern of different mitochondrial lineages (Lougheed *et al.*, 2013; Campagna *et al.*, 2014).

Classical cytogenetics studies of *Z*. *capensis* have shown that this species has 80 chromosomes (de Lucca, 1974; de Lucca and Rocha, 1985). Polymorphisms involving two autosomal pairs were also identified, probably due to intrachromosomal rearrangements, such as inversions (de Lucca and Rocha, 1985; Rocha *et al.*, 1990). The polymorphisms described for *Z*. *capensis* occur in autosomal pairs 2 and 4. Pair 2 can be acrocentric or submetacentric (2^A^ and 2^Sm^), while pair 4 can be acrocentric or metacentric (4^A^ and 4^M^) (de Lucca and Rocha, 1985; Rocha *et al.*, 1990). The presence of these two polymorphic pairs, with the occurrence of four chromosomal forms, allows the combination of nine different cytotypes. All possible cytotypes were found and analyzed, but a geographically structured karyotypic distribution was not found, although there is an apparent positive correlation between the increase in frequency of the 4^M^ form and high latitudes and low temperatures (Carvalho and Erdtmann, 1987; Souza and de Lucca, 1988, 1991; Rocha *et al.*, 1990).

Chromosomal polymorphisms caused by inversions were identified in other bird species in different macrochromosomes; for example, involving pair 1 in *Vanellus vanellus* (Hammar, 1970), pairs 2 and 5 in species of the genus *Junco* (Shields, 1973) and pair 5 in *Cardinalis cardinalis* (Bass, 1979). The first case of a polymorphism was reported for *Zonotrichia albicollis*, involving both pair 2 and pair 3 (Thorneycroft, 1966). Studies performed with bacterial artificial chromosome (BAC) clones indicate that the polymorphism of *Z. albicollis* chromosome 2 was caused by at least two pericentric inversions. In this species, the polymorphism of pair 2 has been correlated with phenotypic and behavioral variations (Thomas *et al.*, 2008).

The vast majority of passerine species have diploid numbers of approximately 80 chromosomes, without substantial variations in genome structure (Christidis, 1990). Fission, fusion and translocation events are rare, but inversions, especially pericentric, are quite common and are regularly found as fixed differences between species and as segregating polymorphisms within species (Hoffmann and Rieseberg, 2008; Faria and Navarro, 2010; Zhang *et al.*, 2014).

The application of comparative chromosome painting using *Gallus gallus* (GGA) probes allows the identification of homologous syntenic blocks that are conserved in the karyotypes of birds (Griffin *et al.*, 2007). In relation to the presumed ancestral karyotype of birds, the Passeriformes present a fission of the first ancestral chromosome (GGA1) (Guttenbach *et al.*, 2003; Dersujeva *et al.*, 2004; Itoh and Arnold, 2005; de Oliveira *et al.*, 2006; Nanda *et al.*, 2011; Kretschmer *et al.*, 2014, 2015; dos Santos *et al.*, 2015, 2017). The use of GGA probes has proven to be efficient in detecting interchromosomal rearrangements, but they are not very informative regarding intrachromosomal rearrangements and cannot indicate the origin of chromosomal breaks (Guttenbach *et al.*, 2003). On the other hand, the use of probes derived from *Leucopternis albicollis* (LAL) has allowed the identification of sites of evolutionary chromosomal breaks (de Oliveira *et al.*, 2010; Kretschmer *et al.*, 2014) as well as of complex rearrangements of chromosomes corresponding to GGA1q that result in paracentric and pericentric inversions (Kretschmer *et al.*, 2014, 2015; dos Santos *et al.*, 2015, 2017). Therefore, LAL probes can be used to generate hypotheses about the mechanisms responsible for these rearrangements.

Although the chromosomal polymorphisms and the karyotype of *Z*. *capensis* (ZCA) have been well characterized via classical cytogenetics, the chromosomal mechanisms that caused these rearrangements remain unknown. Thus, in this work, we used molecular cytogenetic techniques to analyze the karyotypes of three individuals of *Z*. *capensis* with the objective of understanding the chromosomal organization and identifying intrachromosomal rearrangements, as well as comparing the ZCA karyotype with data obtained from other species of Passeriformes.

## Material and Methods

Skin biopsies were obtained from three specimens of *Z*. *capensis* collected in São Gabriel (30°20’45.32” S and 54°19’19.55” W), Rio Grande do Sul State, Brazil. The collections were carried out with the permission of the responsible environmental agency (SISBIO n° 49950-1). The experiments followed protocols approved by the Animal Use Ethics Committee of the Universidade Federal do Rio Grande do Sul (project 29745).

The chromosomes were obtained from cell cultures of fibroblasts according to Sasaki *et al.* (1968). The protocol includes treatment with colchicine (0.05%, 1 h, 37 °C), followed by treatment with hypotonic solution (KCL 0.075 M, 15 min, 37 °C) and fixation with methanol/glacial acetic acid (3:1). Metaphases were conventionally stained (5% Giemsa in 0.07 M phosphate buffer, pH 6.8) for observation of the morphology of the macrochromosomes and verification of the polymorphisms of pairs two and four.

Fluorescent *in situ* hybridization (FISH) with biotin-labeled ribosomal (18S) probes was used for the detection of ribosomal genes, following a protocol described by Daniels and Delany (2003). Chromosome painting was performed with probes of the first ten pairs of GGA chromosomes and with corresponding probes for LAL, GGA1 (LAL3, 6, 7, 15, and 18), GGA2 (LAL2, 4, and 20), GGA3 (LAL9, 13, 17, and 26), GGA4 (LAL1 and 16), GGA5 (LAL5), and GGA6 (LAL3), according to de Oliveira *et al.* (2010). FISH images were photographed through the 63 immersion objective on a Zeiss Imager2 fluorescence microscope and analyzed with AxioVision 4.8 software (Zeiss, Germany).

## Results

**Figure 1:**
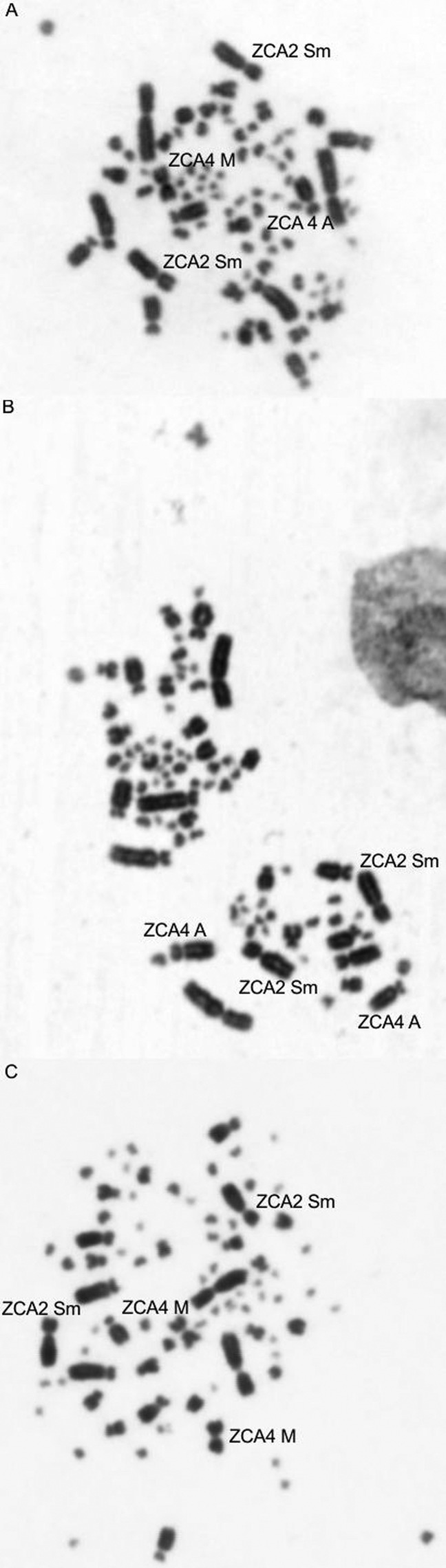
Metaphases of three individuals of rufous-collared sparrow showing the occurrence of a polymorphism in pair 4, which has metacentric or acrocentric morphology. (A) Male: metacentric-acrocentric (B) Male: acrocentric-acrocentric, (C) Female: metacentric-metacentric. The arrows indicate the chromosomes of pair 2 (the species shows polymorphisms in this pair, according to the literature, but in the sampled individuals, we observed only the submetacentric morphology) and pair 4.

The *Z*. *capensis* genome is organized into 80 chromosomes. The first and second pairs are submetacentric, the third and fifth to eighth acrocentric, and ninth and tenth metacentric. The fourth pair is polymorphic, acrocentric or metacentric. The Z sex chromosome is submetacentric, and the W chromosome is metacentric. The remaining chromosome pairs are microchromosomes (Figure 1).

**Figure 2:**
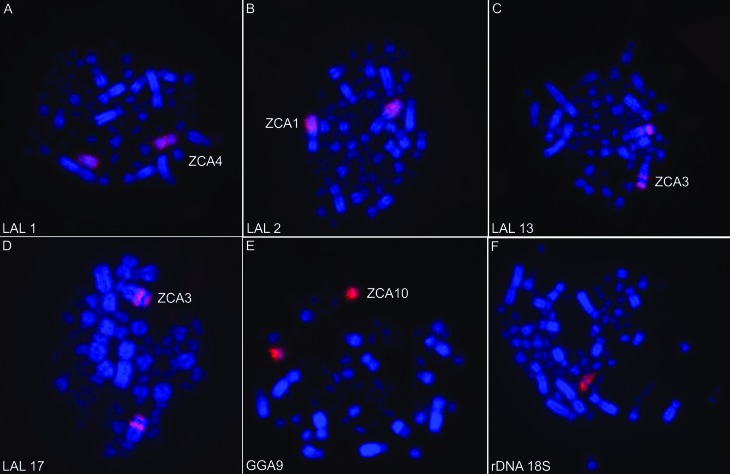
Chromosome hybridization patterns with *Leucopternis albicollis* (LAL) probes (A: LAL1; B: LAL2; C: LAL13; D: LAL17), *Gallus gallus* (GGA) probes (E: GGA9) and 18S rDNA probes (F) onto rufous-collared sparrow metaphases.

**Figure 3:**
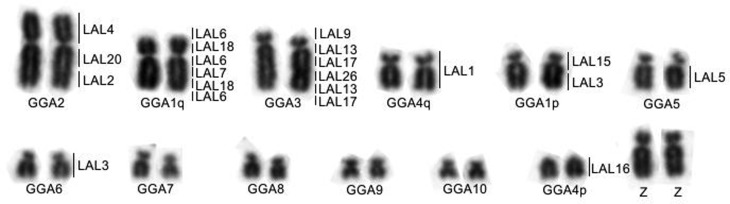
Partial karyotype of the first 12 autosomal pairs of a male (ZZ) indicating the correspondences with *Gallus gallus* (lower) and *Leucopternis albicollis* (right).

Chromosome painting with *Gallus gallus* probes shows the conservation of ancestral macrochromosomes, with the exception of the chromosome GGA1, which corresponds to two pairs, as in all Passeriformes analyzed to date (Figure 2). *Leucopternis albicollis* probes confirm the results obtained with *G. gallus* probes (Figure 2). 18S rDNA probes hybridized to one pair of microchromosomes (Figure 2). The homology map is shown in Figure 3.

**Figure 4:**
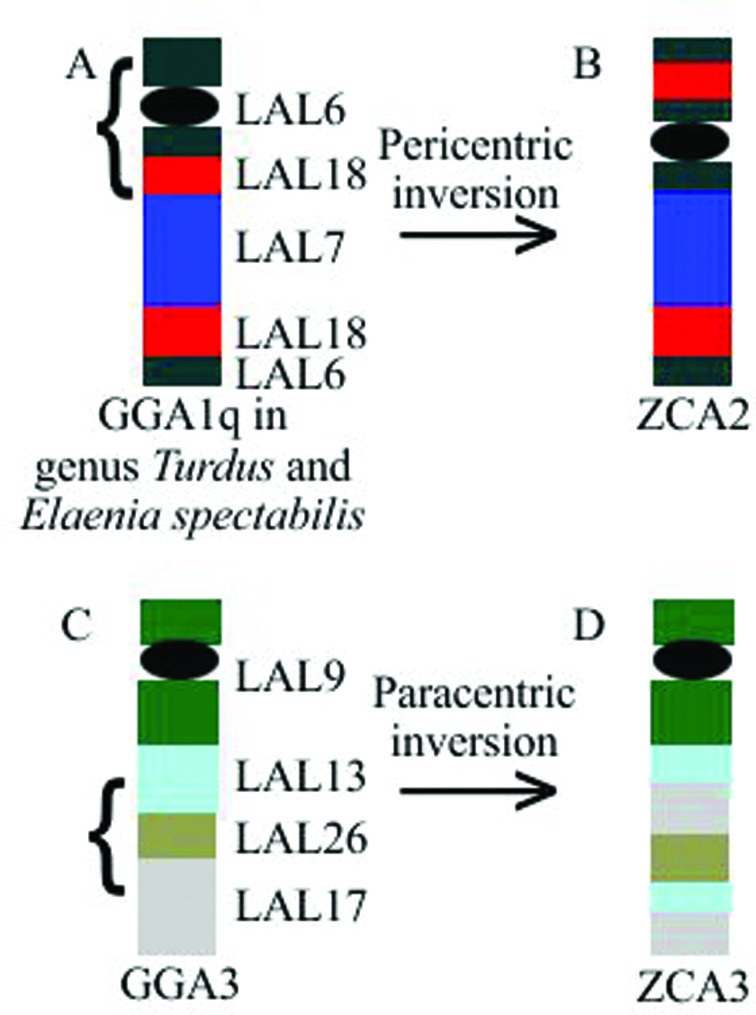
Inversions on the chromosomes of rufous-collared sparrow demonstrated by the application of probes of *L. albicollis*, compared to other passerines for chromosome ZCA2 and compared to GGA3 for chromosome ZCA3.

In adition, *Leucopternis albicollis* chromosome painting allowed the identification of a series of inversions in the chromosome ZCA2 (GGA1q) and one inversion in the chromosome ZCA3 (GGA3). In ZCA2, we observed a break between the fragments of LAL18 and LAL7 and another in the half of LAL6 present in the short arm, leading to a pericentric inversion of the fragment (Figure 4 A,B). In ZCA3 we also observed two breaks, one in LAL13 and one in LAL17 that was followed by a paracentric inversion of the fragment (Figure 4 C,D).

## Discussion

The diploid number of *Zonotrichia capensis* is typical of the order Passeriformes, in which the most common number of chromosomes is 76–80, with few macrochromosomes and many microchromosomes (Christidis, 1990; Kretschmer *et al.*, 2014; dos Santos *et al.*, 2015). The chromosomal morphologies are also similar to those from other passerine species described in the literature. It is known that the second and fourth chromosome pairs are polymorphic in this species (de Lucca and Rocha, 1985). However, in the individuals we sampled, we found polymorphisms only in the fourth pair (metacentric or acrocentric).

The ribosomal sites are in only one pair of microchromosomes, as shown by hybridization of the 18S rDNA probe. This is probably an ancestral character, since all paleognath species (Ratites) have this characteristic (Nishida-Umehara *et al.*, 2007). Other passerine species have ribosomal genes in one pair (*Taeniopygia guttata* and *Saltator* genus), two pairs (*Serinus canaria* and *Turdus albicollis*), or three pairs (*Turdus rufiventris*) of microchromosomes (Kretschmer *et al.*, 2014; dos Santos *et al.*, 2015, 2017). Most likely, in species with more than one pair of microchromosomes with these sequences, duplication of rDNA clusters has occurred with redistribution by translocation (Stitou *et al.*, 1997) or by the action of mobile genetic elements, as reported in plants (Raskina *et al.*, 2008).

Chromosome painting with *Gallus gallus* probes shows the conservation of most of the ancestral macrochromosomes, with the exception of GGA1, which corresponds to two distinct pairs (ZCA2 and ZCA5). The centric fission of the chromosome corresponding to GGA1 has been found in all Passeriformes studied by chromosome painting to date (17 evaluated so far, including ZCA), reinforcing the idea that this was a characteristic present in the last common ancestor of the Passeriformes (Guttenbach *et al.*, 2003; Derjusheva *et al.*, 2004; Itoh and Arnold, 2005; Nanda *et al.*, 2011; Kretschmer *et al.*, 2014, 2015; dos Santos *et al.*, 2015, 2017).

Like GGA probes, LAL probes confirm the fission of putative avian ancestral chromosome 1 and the conservation of the other macrochromosomes. In addition, these probes revealed inversions (paracentric and pericentric) in the ZCA2 chromosome (GGA1q), as in other Passeriformes (Kretschmer *et al.*, 2014, 2015; dos Santos *et al.*, 2015, 2017). However, the order of LAL segments corresponding to GGA1q was different from those found in the other Passeriformes, probably due to an extra inversion. The main difference observed was the presence of a LAL18 fragment in the short arm in *Z*. *capensis*, which has not yet been observed in any other passerine (Kretschmer *et al.*, 2014, 2015).

Chromosome ZCA2 is probably homologous to chromosome 3 of *Z*. *albicollis* (ZAL3) and these two species may share inversions on the same chromosome. It is known that chromosome ZAL2 corresponds to GGA3 (Thomas *et al.*, 2008), but the correspondence between the other ZAL and chicken macrochromosomes is unknown. In all oscine passerines analyzed to date, the first autosomal pair corresponds to GGA2 (Guttenbach *et al.*, 2003; Derjusheva *et al.*, 2004; Itoh and Arnold, 2005; Kretschmer *et al.*, 2014, 2015; dos Santos *et al.*, 2015, 2017), therefore, chromosome ZAL3 of *Zonotrichia albicollis* probably corresponds to GGA1q. In *Zonotrichia capensis*, we considered chromosome ZCA3 as GGA3 and ZCA2 as GGA1q. Because ZCA2 corresponds to ZAL3, the submetacentric form seems to be ancestral for the genus, as hypothesized by Thorneycroft (1975). The observed inversions may have arisen in the ancestor of these species and been maintained in these two strains. However, more studies are needed to evaluate this hypothesis, mainly by mapping BAC clones. There are currently no studies evaluating the polymorphisms in ZAL3.

Although both *Z. albicollis* and *Z. capensis* have inversions on the chromosome corresponding to GGA3 (ZAL2 and ZCA3, respectively), this inversion in *Z*. *capensis* does not lead to the observed polymorphism. Interestingly, none of the seven species of passerine analyzed to date with *Leucopternis albicollis* probes (Kretschmer *et al.*, 2014, 2015, dos Santos *et al.*, 2015, 2017) show inversions in the chromosomes corresponding to GGA3. Most probably, further intrachromosomal rearrangements have occurred in this chromosome after the divergence of these species. Alternatively, both species could have shared intrachromosomal rearrangements, because rearrangements on the chromosome that corresponds to GGA3 are not common in passerine species.

With regard to ZCA4, at the moment it is not possible to indicate by chromosome painting alone that the polymorphism on this chromosome was caused by a pericentric inversion or by centromere repositioning, because this chromosome corresponds to an entire chromosome in *Leucopternis albicollis* (LAL1). In avian, intrachromosomal rearrangements are very common and have been identified *in silico* data (Aslam *et al.*, 2010; Volker *et al.*, 2010; Skinner and Griffin, 2012) and by chromosome painting using *L*. *albicollis* probes (Kretschmer *et al.*, 2014, 2015; dos Santos *et al.*, 2015, 2017). The surprisingly high number of intrachromosomal rearrangements in birds is likely due to the reuse of breakpoints, as previously proposed by Skinner and Griffin (2012). These breakpoint regions show particular enrichment of pseudogenes, long terminal repeats, DNA transposons and long interspersed elements (LINEs) (Skinner and Griffin, 2012; Zhang *et al.*, 2014). However, centromere repositioning has also been reported in birds. The most interesting case is that observed in a comparative study of *G*. *gallus* and *Alectoris rufa* (ARU), as chromosome 4 is submetacentric in *G*. *gallus* and acrocentric in *A*. *rufa*. Previous studies have argued that this difference was due to a pericentric inversion. However, the use of BAC clones for this chromosome showed that the order of the genes is the same in both species, indicating the occurrence of a neocentromere during divergence (Kasai *et al.*, 2003). In *Z*. *capensis,* chromosome 4 is homologous to GGA4q and ARU4q, so it is possible that there is a similar explanation for the rearrangement in ZCA. Breakpoints on this chromosome may have been reused independently in ZCA and in Galliformes.

In summary, we have demonstrated the occurrence of a series of inversions in ZCA2 (GGA1q), as previously proposed for other species of Passeriformes. However, the order of LAL segments in ZCA is different from those in all other species analyzed so far, mainly due to the presence of a fragment of LAL18 in the short arm, which we report for the first time in Passeriformes. We also observed a paracentric inversion in ZCA3, which was previously described in *Z. albicollis*. The presence of polymorphism of chromosome 2 (homologous to ZCA3) in *Z. albicollis* is associated with phenotypic and behavioral variations (Thomas *et al.*, 2008), although a study of the distribution of the different cytotypes (2^A^ and 2^Sm^, 4^A^ and 4^M^) in populations in Brazil did not find a correlation between these polymorphisms and such variations (Souza and de Lucca, 1991). Therefore, further investigations sampling the polymorphism of pair 2 among the numerous subspecies of *Zonotrichia capensis*, as well as using techniques with more refined screening of inversions, such as a BAC cloning map, could shed light on possible relationships between chromosomal polymorphism and the phenotypic differences reported throughout the species distribution.
